# Genesis or Evolution of Gender Differences? Worldview-Based Dilemmas in The Processing of Scientific Information

**DOI:** 10.5334/joc.99

**Published:** 2020-04-30

**Authors:** Stephan Lewandowsky, Jan K. Woike, Klaus Oberauer

**Affiliations:** 1 University of Bristol, GB; 2 University of Western Australia, AU; 3 Max Planck Institute for Human Development, DE; 4 University of Zurich, CH

**Keywords:** Emotion and cognition, Social cognition, Reasoning

## Abstract

Some issues that have been settled by the scientific community, such as evolution, the effectiveness of vaccinations, and the role of CO_2_ emissions in climate change, continue to be rejected by segments of the public. This rejection is typically driven by people’s worldviews, and to date most research has found that conservatives are uniformly more likely to reject scientific findings than liberals across a number of domains. We report a large (N > 1,000) preregistered study that addresses two questions: First, can we find science denial on the left? Endorsement of pseudoscientific complementary and alternative medicines (CAM) has been anecdotally cited as being more consonant with liberals than conservatives. Against this claim, we found more support for CAM among conservatives than liberals. Second, we asked how liberals and conservatives resolve dilemmas in which an issue triggers two opposing facets of their worldviews. We probed attitudes on gender equality and the evolution of sex differences—two constructs that may create conflicts for liberals (who endorse evolution but also equality) and conservatives (who endorse gender differences but are sceptical of evolution). We find that many conservatives reject both gender equality and evolution of sex differences, and instead embrace “naturally occurring” gender differences. Many liberals, by contrast, reject evolved gender differences, as well as naturally occurring gender differences, while nonetheless strongly endorsing evolution.

There is no scientific debate about the fact that all species, including humans, evolved by a process of natural selection (Pew Research Center, 2015). There is no debate in the medical community about the vast improvement to public health that has resulted from widespread childhood vaccinations (Whitney, Zhou, Singleton, & Schuchat, 2014). There is also overwhelming evidence that many forms of complementary and alternative “medicine” (CAM), such as homeopathy, are ineffective. Reliance on CAM can even lead to unnecessary deaths if it causes cancer patients to refuse or delay evidence-based treatment (Johnson, Park, Gross, & Yu, 2018).

The scientific consensus for evolution and vaccinations, and against complementary “medicine”, stands in sharp contrast to the opinions of a sizable segment of the public. For example, whereas 98% of scientists accept that humans evolved over time, only 65% of the American public shares that view (Pew Research Center, 2015). Similarly, whereas 86% of scientists believe that childhood vaccinations should be mandatory, this view is only shared by 68% of the public (Pew Research Center, 2015). The presence of contrarian public opinions, even if they are only held by a minority, can have adverse consequences: anti-vaccination movements have had discernible impact on public health (Gangarosa et al., 1998; Smith, Yarwood, & Salisbury, 2007) and organizations that oppose evolution have undermined science curricula in many American school districts (Watts, Hossfeld, Tolstikova, & Levit, 2017).

In consequence, there has been increasing research interest in the variables that explain why people reject scientific facts. Two consistent findings have emerged from this research: First, educational attainment, scientific knowledge, and science literacy are at best modestly predictive of attitudes concerning scientific issues (e.g., Allum, Sturgis, Tabourazi, & Brunton-Smith, 2008; Tom, 2018). Second, people’s worldviews, that is their deeply-held beliefs about the world and how society should be organized, have been identified as the preeminent predictor of attitudes towards scientific evidence across numerous topics. In particular, in American participants, the rejection of science is principally associated with rightwing or libertarian worldviews. Whether it is climate change (e.g., Hamilton, 2011; Hamilton, Hartter, Lemcke-Stampone, Moore, & Safford, 2015; Lewandowsky, Gignac, & Oberauer, 2013), vaccinations (e.g., Hamilton, Hartter, & Saito, 2015; Kahan, Braman, Cohen, Gastil, & Slovic, 2010; Lewandowsky, Gignac, & Oberauer, 2013), evolution (e.g., Hamilton, 2015; Tom, 2018), genetically-modified organisms (e.g., Hamilton, 2015), or even nuclear energy (e.g., Hamilton, 2015), people on the political left trust scientists more on those issues and tend to accept the pertinent scientific findings more than their counterparts on the political right.

Two important questions, however, remain unresolved: first, are there any domains in which the role of worldviews is reversed—that is, do American liberals reject well-established scientific findings that conservatives endorse? Second, how do people respond to situations in which their worldview provides conflicting imperatives that are not readily reconcilable? The present study was designed to address these two questions.

## Attitudinal symmetry and scientific evidence

In many cases, the association between rightwing worldviews and rejection of scientific evidence is easy to understand. For example, climate change is a direct consequence of fossil-fuel powered economic growth, and successful climate mitigation will require cuts to greenhouse gas emissions (e.g., Knutti & Rogelj, 2015) that are not achievable without massive restructuring of the global economy and large-scale deployment of new technologies (Anderson & Peters, 2016). Accepting the existence and origins of climate change is therefore tantamount to accepting that unregulated markets can create problems whose solution requires state intervention—clearly a challenging proposition for many conservatives and libertarians. Similarly, libertarians may oppose public-health measures, such as mandatory childhood vaccinations, because they constitute government intervention (Kahan et al., 2010). Evolution is typically opposed for religious reasons (Mazur, 2004; J. D. Miller, Scott, & Okamoto, 2006; Tom, 2018), and via the association between religiosity and rightwing worldviews (Malka, Lelkes, Srivastava, Cohen, & Miller, 2012), this opposition will also express itself when worldviews are measured to predict attitudes towards evolution.

If conservatives’ rejection of science arises because the evidence challenges their political worldviews, then liberals might likewise adopt problematic attitudes or reasoning strategies when scientific evidence runs counter to their own worldviews. Previous attempts to discover scientific propositions that are rejected by the political left have focused on genetically-modified organisms (GMO) and vaccinations, based largely on anecdotal media reports that claimed left-wing opposition to GMO foods (e.g., Shermer, 2013) and vaccinations (e.g., Mooney, 2011). Those suggestions have not withstood scrutiny (Hamilton, 2015; Lewandowsky, Gignac, & Oberauer, 2013; Rabinowitz, Latella, Stern, & Jost, 2016).^1^

Here we continue our search for science denial on the left by examining attitudes towards complementary and alternative medicine (CAM). CAM is particularly suitable for this search because of anecdotal claims that alternative medicine and vaguely left-wing ideas may have found a symbiotic home under the “New Age” umbrella (see, e.g., Keshet, 2009). Sociologists have also linked CAM use to “resistance” movements, such as antipharmaceutical activism or community development (Gale, 2014). Homeopathy, for example, has been cited as “feminist medicine” (Scott, 1998). On the basis of this largely qualitative research one might expect people on the political left to be more hesitant to reject CAM despite the lack of scientific evidence supporting it than people on the right.

## Gender equality vs. differences

One of the core tenets of liberalism is the belief in the capacity to improve people and their circumstances. This belief, often known as meliorism, is at the heart of liberalism (e.g., Castagno, 2017; Porter, 2013), whereas disbelief or skepticism in that possibility characterizes conservatives. Belief in the possibility of general human improvement is thus higher among liberals than conservatives (L. Miller & Seligman, 1999). One long-standing and strong implication of liberal meliorism is belief in gender equality. Assuming that no important difference between the sexes is given by nature makes it easier to argue that all existing differences can be remedied by societal reform. Conservatives, by contrast, reject this possibility and may therefore be more likely to find genders to be ineluctably unequal. Indirect evidence for this hypothesis comes from a large body of literature that has found strong associations between conservatism and sexism (e.g., Hodson & MacInnis, 2017), and between other indicators of rightwing politics such as Rightwing Authoritarianism or social dominance orientation and sexism (e.g., Hellmer, Stenson, & Jylhä, 2018; Van Assche, Koç, & Roets, 2019). However, we know of no work that has examined conservatives’ beliefs concerning the origins of gender differences.

The scientific debate whether nature (i.e., biology, evolution, and genetics) or nurture (i.e., social variables such as parenting and societal stereotypes) has a stronger influence on gender differences has been raging for decades (for a recent review, see Eagly & Wood, 2013). Arguably, some of the positions taken during this debate were shaped and constrained by ideology in addition to data and evidence (Eagly, 2018). The involvement of ideology is unsurprising in light of a long-standing and deep fissure among American feminists and legal scholars between “sameness” and “difference.” Mid-twentieth-century feminism laid claim to the essential equality between men and women by highlighting their fundamental similarity. This emphasis on sameness shifted towards greater recognition of gender differences in the closing decades of the twentieth century (Williams 1989). Both approaches share the goal of achieving gender equality but they pursue quite different strategies. For example, when confronted with the issue of pregnancy in the workplace, the approaches share a common goal—supporting pregnant women—but differ in “whether to stress the similarities between men and women (in order to gain support for pregnant women) or whether (and when) to stress their differences (in order to gain support for pregnant women)” (Cain, 1989, p. 804) At one end of this continuum, scholars search for “feminist insights into women’s true nature” (West, 1988, p. 4). At the other extreme, scholars have replaced this “essentialist” view of women, whether biologically or socially inspired, with radical social constructivism (e.g., MacKinnon, 1989).

Although the scientific nature-nurture debate has not been conclusively resolved (Eagly & Wood, 2013), the available evidence appears to rule out either extreme position. For example, a purely biological invariant essentialism is challenged by the fact that gender-specific mate preferences have changed considerably over the past 50 years. Men increasingly prefer women with good financial prospects whereas housekeeping skills have become less important for choice, and conversely, women increasingly desire men with good looks (e.g., Buss, Shackelford, Kirkpatrick, & Larsen, 2001). Overall, there has been substantial convergence between the sexes in their stated mate preferences during the past few decades. Similarly, a purely constructivist view of gender differences is difficult to reconcile, at first glance, with the fact that the more gender-equal countries are, the *greater* is their gender gap in the number of graduates in science, technology, engineering, and mathematics (Stoet & Geary, 2018). For example, Finland excels in gender equality but has one of the world’s largest gender gaps in science-based college degrees.

For our study, the unresolved scientific status of gender differences shifts emphasis from comparing people’s attitudes to a scientific “gold standard”—as is possible with issues such as evolution, vaccinations, or climate change—to examining how people resolve dilemmas arising from conflicting imperatives of their worldview. It turns out that liberals’ belief in gender equality gives rise to conflicting imperatives that are not easy to resolve. Conservatives are similarly confronted with gender-related dilemmas, albeit of a different nature.

## Conflicting imperatives of worldview

The fact that humans evolved is widely accepted by people on the political left, and rejected by some on the right (Tom, 2018). Acceptance of evolution creates a potential dilemma for liberals, given that some evolutionary psychologists have been instrumental in drawing scientific attention to ostensibly biologically-determined gender differences, such as mate choice (e.g., Buss, 1989).^2^ “To an evolutionary psychologist, the likelihood that the sexes are psychologically identical in domains in which they have recurrently confronted different adaptive problems over the long expanse of human evolutionary history is essentially zero” (Buss, 1996, p. 301). How, then, will liberals reconcile their acceptance of Darwinian evolution with its potential detrimental impact, by some interpretations, on another cherished aspect of liberalism, namely meliorism and its tacit acceptance of gender equality? Although research on evolved gender differences does not necessarily compromise society’s commitment to gender equality, the invocation of seemingly ineluctable evolutionary factors does provide political actors with opportunities to argue against equality.

We are not aware of any research that has examined this question in the public at large. However, some scholarly attention has focused on how members of scientific disciplines often identified with a liberal orientation—namely, social psychology and sociology—navigate the waters between Darwinian evolution and the implications of a variant of evolutionary psychology that postulates evolved differences in behavior and its neural substrate. Von Hippel and Buss (2017) asked a sample of more than 300 social psychologists about evolution and gender differences (and other traits and behaviors not relevant here). The results showed that the sampled scientists overwhelmingly accepted the theory of evolution, providing a mean rating of 88% on a scale from 0–100% that Darwin’s ideas are likely to be true. By contrast, the mean ratings for the propositions that “women’s brains evolved to be more verbally talented” and that “men’s brains evolved to be more mathematically talented” were at 40% and 30%, respectively. In another survey of sociologists, Horowitz, Yaworsky, and Kickham (2014) found that 43% of respondents found it plausible or highly plausible that “differences between women and men in such skills as communication and spatial reasoning are linked to biological differences in female and male brains”. A further 22% were undecided and only 35% found this possibility implausible. Moreover, Horowitz et al. found that self-identified feminist theoreticians were less likely to endorse biological-evolutionary factors as underpinning social behavior than sociologists with a different theoretical orientation. (See Legare, Opfer, Busch, & Shtulman, 2018, for a detailed exploration of the fit between evolution and the social sciences.)

Overall, the available data suggest that although scientists generally accept the importance of nature in explaining gender differences, they are more skeptical of claims linking evolved differences in brain structure to gender differences in behavior. This skepticism can draw justification from recent work in neuroscience that has emphasized the plasticity of human brains as well as the fluidity of gender differences (e.g., Fine, 2013; Fine, Jordan-Young, Kaiser, & Rippon, 2013; Fine, Joel, Jordan-Young, Kaiser Trujillo, & Rippon, 2014). Recent research on neuroplasticity may thus point to a resolution of the dilemma for scientists, but it remains to be seen how members of the public respond to the same dilemma.

The reverse dilemma confronts conservatives: their pervasive preference to reject gender equality could be buttressed by appealing to evolved, biological gender differences. However, any such appeal would require at least tacit acceptance of Darwinian evolution, which would also be conflicting for many conservatives. How will conservatives reconcile their reluctance to embrace evolution with its potential utility in buttressing another cherished aspect of conservatism, namely its endorsement of immutable gender differences?

Our study explored these worldview-based dilemmas for liberals and conservatives by measuring three different constructs relating to gender differences. We measured people’s beliefs about men and women being the same in all respects, men and women having evolved differently, and men and women being “naturally different.” The latter two constructs both allowed for an endorsement of gender differences, but in one case those differences were presented as the result of evolution, whereas in the other case those differences were presumed to exist “naturally” without specifying any particular cause (e.g., evolution, divine intervention). By remaining ambiguous about the mechanism underlying gender differences, the latter construct avoids triggering worldview-based opposition to any particular means by which gender differences may have arisen (e.g., evolution or divine intervention).

**Figure 1:**
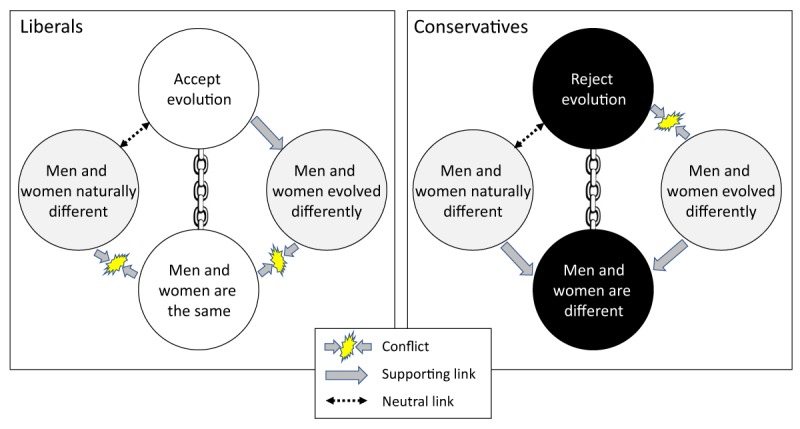
Overview of the dilemmas facing liberals (left panel) and conservatives (right panel) in the context of gender differences and evolution. Core beliefs are shown in white (liberals) and black (conservatives) to reflect their opposing polarity. Because core beliefs are thought to be inviolable they are connected by chains. Potential explanatory variables for gender differences are shown in gray. Arrows represent presumed conflict between constructs, support, or neutrality.

Figure 1 illustrates the relationship between our gender constructs, and participants’ presumed core beliefs. For liberals, we expected endorsement of Darwinian evolution and gender equality to constitute core beliefs. Conversely, for conservatives, rejection of evolution and rejection of gender equality were expected to constitute core beliefs. The conflicts indicated in the figure follow from those core beliefs: although evolved gender differences would explain why men and women are different, this would be in conflict with conservatives’ rejection of evolution. Conversely, for liberals the idea of evolved differences fits well with acceptance of evolution but is in potential conflict with gender equality.

At least two possible resolutions of those dilemmas can be anticipated: First, people might recruit the explanatory construct (i.e., evolved gender differences) to support their core attitudes about gender. Thus, conservatives might accept evolved gender differences because they buttress their belief in differences between men and women, and liberals might reject evolved gender differences because no such differences are presumed to exist. This resolution would create conflict for both groups regarding their core attitudes towards evolution. The second resolution would involve the reverse: both groups ensure that their attitudes involving evolution are internally consistent, with liberals endorsing evolved gender differences and conservatives rejecting them. This resolution would create conflict for both groups regarding their core attitudes towards gender differences instead. Neither resolution, however, can entirely resolve the conflict posed by our competing constructs.

## Method

### Overview

We conducted a representative survey of 1,000 American residents. Our results and conclusions are therefore specific to the American cultural context. The survey measured 10 constructs that belonged to 5 conceptual groups: (1) We measured people’s endorsement of two scientific constructs, namely evolution and vaccinations. (2) We also measured people’s attitudes towards one instance of pseudoscience, namely complementary and alternative medicines (CAM). To facilitate comparison with the other scientific constructs, we coded this construct to represent *rejection* of CAM (and hence acceptance of the scientific view that CAM is ineffective). (3) We measured people’s worldviews via three related but distinct constructs; namely, religiosity, socio-political conservatism, and endorsement of free markets. (4) We created three constructs related to gender differences (Figure 1). (5) The final construct comprised the three questions from the cognitive reflection test (CRT; Frederick, 2005). The CRT measures people’s propensity to engage in analytical reasoning. CRT performance has been previously identified as a strong predictor of science understanding (Shtulman & McCallum, 2014), and we therefore expect it to predict acceptance of science, and rejection of pseudoscience, in our study as well.

The sampling plan and procedure as well as an analysis plan were preregistered before data collection commenced. The preregistration document, including a complete copy of the survey can be found on *GitHub* at https://git.io/fjpeB.

### Materials

The survey comprised 60 items, broken down into 2 demographic queries presented at the outset (age and gender); 14 items involving a “slider” scale (0–100) for the socio-political conservatism construct; 40 items on a 7-point scale (from “Strongly Disagree” to “Strongly Agree”) to measure our remaining core attitudinal constructs; the 3 items of the CRT; and 1 item that served as attention filter. The attention filter asked people to identify which of a list of 5 candidates was not an animal.

The socio-political conservatism items were taken from Everett (2013) (the full set reported in Table 1) and asked participants to indicate “the extent to which you feel positive or negative towards an issue”, with 50 taken to be the neutral point and 0 representing great negativity and 100 great positivity, respectively. The 14 issues probed (and, where applicable, their short labels used for presentation of the results) were Abortion, Welfare benefits (*Welfare*), Tax, Immigration, Limited government (*LimGov*), Military and national security (*Military*), Religion, Gun ownership (*Guns*), Traditional marriage (*TradMar*), Traditional values (*TradVal*), Fiscal responsibility (*FiscResp*), *Business*, the family unit (*Family*), and *Patriotism*. Each participant received the 14 slider scales in a uniquely-generated random order.

**Table 1:** Items with 7-point response scale used in the survey and their short names.

Item name	Item (R = reverse scored)

1. Free market	

*FMUnresBest*	An economic system based on free markets unrestrained by government interference automatically works best to meet human needs.
*FMLimitSocial*	The free market system may be efficient for resource allocation but it is limited in its capacity to promote social justice. (R)
*FMMoreImp*	The preservation of the free market system is more important than localized environmental concerns.
*FMThreatEnv*	Free and unregulated markets pose important threats to sustainable development. (R)
*FMUnsustain*	The free market system is likely to promote unsustainable consumption. (R)
2. Religiosity	

*RelComf*	Do you agree with the following statement? “Religion gives me a great amount of comfort and security in my life.”
*RelGod*	I believe in God.
*RelAfterlife*	I believe in some kind of afterlife.
*RelNatWorld*	I do not think religion can or should make claims about the natural world. (R)
*RelRelig*	I do not consider myself a religious person. (R)
3. Evolution	

*EvoAnimals*	I believe that animals have changed over time by a process of evolution.
*EvoSupported*	I accept evolution by natural selection as a well-supported scientific theory.
*EvoSpecies*	I believe that all species, including humans, have a common evolutionary origin.
*EvoCreated*	I believe that species were created individually and do not change over time. (R)
*EvoCrisis*	I believe that the theory of evolution by natural selection is in crisis and about to be overturned. (R)
4. Vaccinations	

*VaxSafe*	I believe that vaccines are a safe and reliable way to help avert the spread of preventable diseases.
*VaxNegSide*	I believe that vaccines have negative side effects that outweigh the benefits of vaccination for children. (R)
*VaxTested*	Vaccines are thoroughly tested in the laboratory and wouldn’t be made available to the public unless it was known that they are safe.
*VaxRisky*	The risk of vaccinations to maim and kill children outweighs their health benefits. (R)
*VaxContrib*	Vaccinations are one of the most significant contributions to public health.
5. Rejection of Complementary and alternative medicine (CAM)	

*CAMDanger*	Complementary medicine can be dangerous in that it may prevent people getting proper treatment.
*CAMCure*	Complementary medicine builds up the body’s own defenses, so leading to a permanent cure. (R)
*CAMIneffect*	Homeopathy has been shown again and again to be ineffective as a cure for anything.
*CAMSaves*	Complementary medicine has often saved the lives of patients who were already given up by conventional doctors. (R)
*CAMSuperior*	Complementary medicine is superior to conventional medicine in treating chronic ailments such as allergies, headaches, and back pains. (R)
6. Men and women evolved differently	

*MWEvoDiff*	Men and women evolved to be different and these biological differences cannot be overcome by education.
*MWEvoViol*	Evolutionary history has predisposed men more strongly than women towards violence.
*MWEvoNurture*	Evolutionary history has predisposed women more strongly than men towards being helpful and nurturing.
*MWEvoTraits*	All human traits are the product of evolution and therefore resist change.
*MWEvoDiff2*	Thousands of years of evolution explain why differences between men and women are very difficult to overcome.
7. Men and women are naturally different	

*MWNatStrong*	Men are naturally stronger than women and those differences cannot be overcome by education.
*MWNatAggress*	It is the in the nature of men to be physically aggressive more often than women.
*MWNatCaring*	Women are naturally more caring and socially supportive than men.
*MWNatTraits*	All human traits are part of our natural makeup and therefore very difficult to change.
*MWNatDiff*	Men and women are naturally different from each other and those differences are bound to stay, even if we try hard to overcome them.
8. Men and women are the same	

*MWEqu*	Men and women are equally capable and powerful in all respects.
*MWEquDiff*	All differences between men and women are created by society and can be eliminated if we change society.
*MWEquCulture*	Without the pressures of culture and society women would be as much in control as men.
*MWEquNoBio*	There are no biological or physical reasons that prevent a girl today to achieve as much as a boy.
*MWEquInvent*	The categories “male” and “female” are primarily cultural inventions that have little basis in human nature.

The 40 items that measured core attitudinal constructs and the attention filter were presented in a different random order for each participant. Table 1 provides a verbatim list of these 40 items together with brief labels (e.g., *FMUnresBest* for “An economic system based on free markets unrestrained by government interference automatically works best to meet human needs”) that are used for presentation of the results.

The items for the three gender-related constructs were designed by the authors for this study. (Pilot testing confirmed that their psychometric properties were satisfactory.) The items for the free-market and vaccination constructs were taken from our earlier research (e.g., Lewandowsky, Gignac, & Oberauer, 2013). The items for the religiosity and evolution constructs were adapted from Lombrozo, Thanukos, and Weisberg (2008). The CAM-rejection construct was probed by taking two items (*CAMDanger* and *CAMCure*) from Hyland, Lewith, and Westoby (2003), and combining them with three other items developed by the authors.

### Ethics statement

The Ethics Committee of the Max Planck Institute for Human Development in Berlin approved the study. The survey was prefixed by an introductory information sheet outlining the research. Participants indicated their informed consent after reading this information sheet by a mouse click, which commenced presentation of the survey questions.

### Participants and procedure

A sample of 1,000 U.S. residents 18 years and older was recruited during June 2018 via electronic invitations by Qualtrics.com, a firm that specializes in representative internet surveys. Participants were drawn from a representative panel of more than 5.5 million U.S. residents (as of January 2013), via propensity weighting to ensure representativeness in terms of gender, age, and income. Participants were compensated by Qualtrics using the company’s standard reward scheme.

As part of the sampling procedure, Qualtrics conducts a “softlaunch” (*N* ≃ 50) that provides an opportunity for inspection of the data. We discovered that numerous participants in this preliminary sample responded identically (before reverse-coding) to all items for one or more constructs. To deal with this indication of inattention, a further attention filter question was inserted as part of the socio-political conservatism slider scale that asked participants to select the value “20”. Upon relaunch, an inspection of a further preliminary sample (*N =* 50) suggested that the additional attention filter solved the inattention problem and hence sampling proceeded for the full quota.

## Results

The final sample, after exclusion of participants from the initial softlaunch, included 1017 responses that passed the Qualtrics quality checks (including the two attention filters). The sample size slightly exceeded the contracted quota of 1,000 because of a brief delay between achieving the quota and shutting down of the survey. This data file (stripped of geo-tagging and other potentially identifying information), the R scripts for all analyses, and the L_A_T_E_X source file that weaves the results of the analysis directly into the paper can be found at https://git.io/fjpL5.

The final sample included 489 men and 528 women, with a mean age of 46.5 years (median 47; range 18–99). Mean age differed between men (52.0) and women (41.4).

### Data summary and adjustment

**Figure 2:**
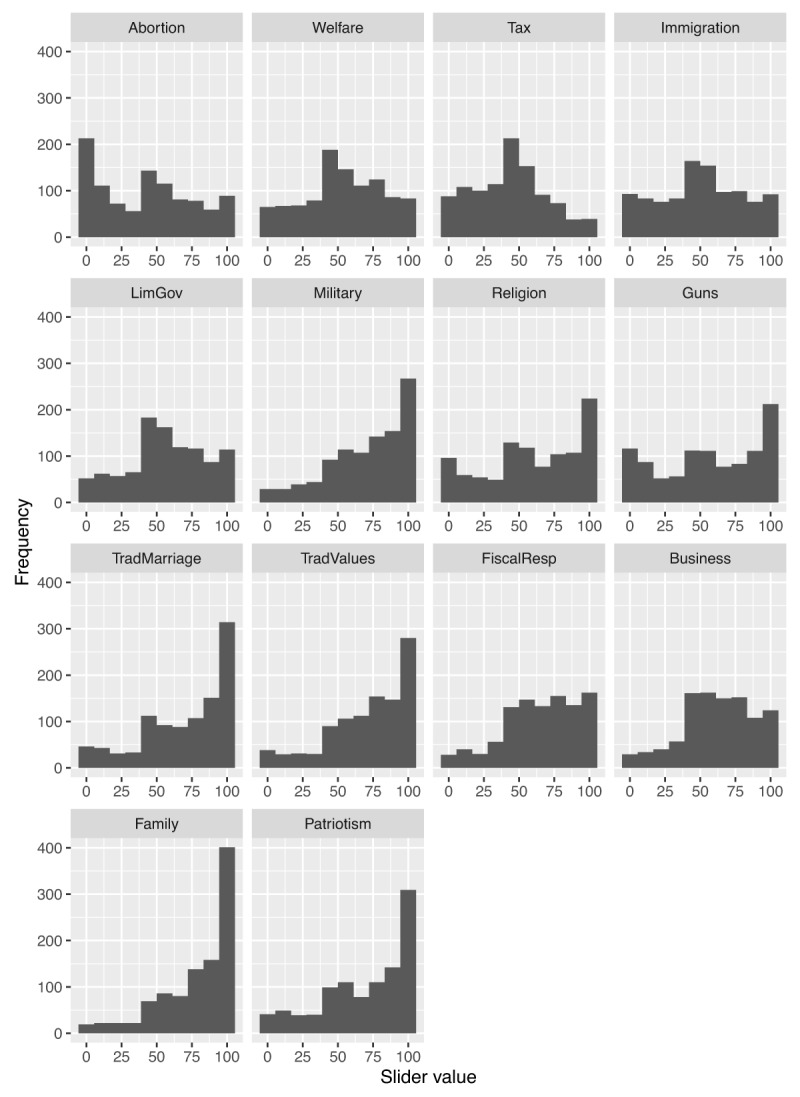
Frequency distributions of responses for the 14 items of the socio-political conservatism scale. Each histogram shows the distribution across subjects of the slider response which ranged from 0 (strong negativity) to 100 (high positivity).

Figure 2 shows the distribution of slider responses to the 14 items of the socio-political conservatism scale. Table 2 shows the number and percentages of responses to the 40 core items before reverse-scoring (for item labels, see Table 1). For the constructs that contained items of varying polarities, the mean of the responses to all reverse-scored items (3.94) was found to be closer to the midpoint of the scale (4) than the mean response to all non-reverse-scored items (4.87), suggesting the presence of an affirmation bias, that is a tendency to respond “yes” to any item regardless of its polarity. Moreover, the magnitude of that affirmation bias (i.e., the difference between the two item sets of different polarity for each participant) varied considerably across individuals. Individual differences in affirmation bias are problematic because they can inflate estimates of correlations between items with the same polarity, and underestimate correlations between items with differing polarity.

**Table 2:** Number of responses (percentages) for each response option for all survey items using a 7-point scale.

Item name	Strongly disagreed		Disagree		Somewhat disagreed		Neither agree nor disagree		Somewhat agree		Agree		Strongly agree	

1. Free market														

FMUnresBest	62	(6)	68	(7)	107	(11)	330	(32)	212	(21)	132	(13)	106	(10)
FMLimitSocial	20	(2)	40	(4)	78	(8)	376	(37)	237	(23)	164	(16)	102	(10)
FMMoreImp	94	(9)	99	(10)	152	(15)	346	(34)	147	(14)	111	(11)	68	(7)
FMThreatEnv	50	(5)	66	(6)	117	(12)	327	(32)	212	(21)	141	(14)	104	(10)
FMUnsustain	49	(5)	78	(8)	114	(11)	379	(37)	179	(18)	134	(13)	84	(8)
2. Religiosity														

RelComf	159	(16)	71	(7)	56	(6)	162	(16)	176	(17)	164	(16)	229	(23)
RelGod	87	(9)	30	(3)	29	(3)	111	(11)	84	(8)	150	(15)	526	(52)
RelAfterlife	59	(6)	34	(3)	28	(3)	146	(14)	122	(12)	231	(23)	397	(39)
RelNatWorld	129	(13)	98	(10)	90	(9)	359	(35)	110	(11)	88	(9)	143	(14)
RelRelig	228	(22)	161	(16)	112	(11)	115	(11)	108	(11)	129	(13)	164	(16)
3. Evolution														

EvoAnimals	56	(6)	29	(3)	45	(4)	137	(13)	214	(21)	280	(28)	256	(25)
EvoSupported	90	(9)	46	(5)	55	(5)	240	(24)	210	(21)	193	(19)	183	(18)
EvoSpecies	80	(8)	43	(4)	54	(5)	206	(20)	205	(20)	240	(24)	189	(19)
EvoCreated	168	(17)	168	(17)	185	(18)	182	(18)	110	(11)	116	(11)	88	(9)
EvoCrisis	104	(10)	94	(9)	123	(12)	382	(38)	152	(15)	97	(10)	65	(6)
4. Vaccinations														

VaxSafe	41	(4)	16	(2)	44	(4)	111	(11)	157	(15)	252	(25)	396	(39)
VaxNegSide	286	(28)	176	(17)	120	(12)	187	(18)	115	(11)	59	(6)	74	(7)
VaxTested	44	(4)	31	(3)	77	(8)	183	(18)	217	(21)	268	(26)	197	(19)
VaxRisky	262	(26)	171	(17)	116	(11)	265	(26)	72	(7)	63	(6)	68	(7)
VaxContrib	27	(3)	10	(1)	40	(4)	158	(16)	195	(19)	285	(28)	302	(30)
5. Complementary and alternative medicine (CAM)														

CAMDanger	33	(3)	41	(4)	101	(10)	332	(33)	249	(24)	166	(16)	95	(9)
CAMCure	46	(5)	61	(6)	129	(13)	434	(43)	188	(18)	94	(9)	65	(6)
CAMIneffect	90	(9)	109	(11)	159	(16)	381	(37)	136	(13)	77	(8)	65	(6)
CAMSaves	18	(2)	17	(2)	41	(4)	381	(37)	264	(26)	183	(18)	113	(11)
CAMSuperior	46	(5)	64	(6)	103	(10)	443	(44)	177	(17)	111	(11)	73	(7)
6. Men and women evolved differently														

MWEvoDiff	104	(10)	103	(10)	144	(14)	208	(20)	176	(17)	159	(16)	123	(12)
MWEvoViol	62	(6)	57	(6)	83	(8)	262	(26)	256	(25)	200	(20)	97	(10)
MWEvoNurture	52	(5)	39	(4)	64	(6)	231	(23)	253	(25)	240	(24)	138	(14)
MWEvoTraits	104	(10)	134	(13)	180	(18)	271	(27)	163	(16)	104	(10)	61	(6)
MWEvoDiff2	86	(8)	77	(8)	97	(10)	282	(28)	243	(24)	141	(14)	91	(9)
7. Men and women naturally differ														

MWNatStrong	100	(10)	79	(8)	126	(12)	192	(19)	205	(20)	178	(18)	137	(13)
MWNatAggress	48	(5)	54	(5)	85	(8)	188	(18)	279	(27)	218	(21)	145	(14)
MWNatCaring	28	(3)	39	(4)	67	(7)	200	(20)	288	(28)	221	(22)	174	(17)
MWNatTraits	50	(5)	80	(8)	144	(14)	221	(22)	254	(25)	170	(17)	98	(10)
MWNatDiff	41	(4)	47	(5)	75	(7)	171	(17)	270	(27)	223	(22)	190	(19)
8. Men and women are equal														

MWEqu	40	(4)	35	(3)	100	(10)	115	(11)	180	(18)	243	(24)	304	(30)
MWEquDiff	108	(11)	111	(11)	161	(16)	199	(20)	188	(18)	129	(13)	121	(12)
MWEquCulture	32	(3)	40	(4)	74	(7)	211	(21)	228	(22)	214	(21)	218	(21)
MWEquNoBio	32	(3)	36	(4)	75	(7)	123	(12)	151	(15)	258	(25)	342	(34)
MWEquInvent	206	(20)	170	(17)	161	(16)	229	(23)	116	(11)	82	(8)	53	(5)

For most constructs, this affirmation bias was at least partially controlled by the inclusion of items of both polarities. However, the three gender-related sets of items did not include any reverse-scored items (with the entire “men and women are the same” cluster instead serving as reverse-polarity items for gender). We therefore controlled the affirmation bias for the gender-related items by statistical means. We defined the mean response across all remaining items (i.e., all clusters other than gender irrespective of polarity) before reverse scoring as a person’s affirmation-bias score. Responses to each of the gender items were then regressed on that affirmation-bias score, and the resulting residuals were added to the mean response for that item to create bias-adjusted responses. This procedure leaves the mean of each gender item across participants unchanged, but replaces each person’s observation with a “true endorsement” score that is obtained by removing his or her individual affirmation bias.

All analyses are based on the affirmation-bias adjusted responses for the three gender constructs. Because this affirmation bias was unexpected, the adjustment could not be preregistered, and all remaining analyses therefore depart from the preregistered analysis plan for the gender constructs. The conclusions from this study are not materially altered if unadjusted responses are used instead. We do not report the unadjusted analysis in this article but all output and figures for the unadjusted analysis can be found in the docs folder at the repository for this article (https://git.io/fjpL5).

**Figure 3:**
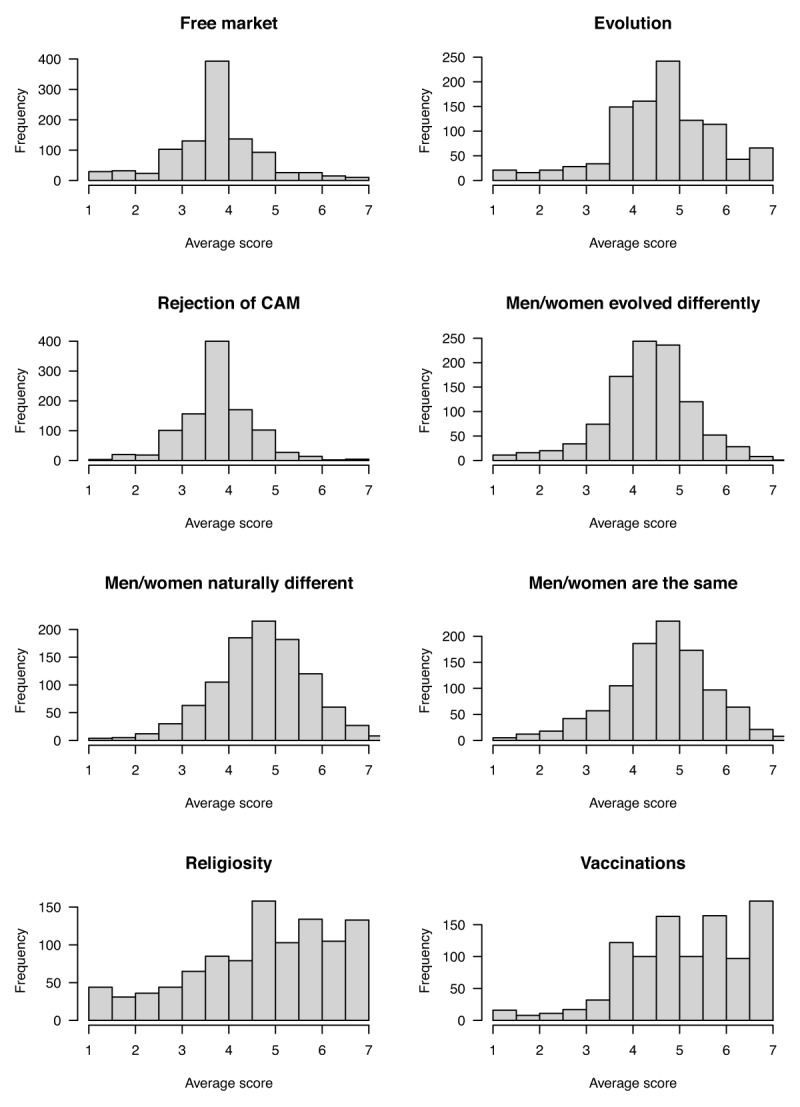
Frequency distributions of the composite scores for all constructs excluding socio-political conservatism, formed by averaging responses across items within each construct after reverse scoring. Each histogram shows the distribution across subjects of the composite score. Table 1 provides an overview of the items for each construct. All items were accompanied by a 7-point response scale ranging from “Strongly agree” (coded as 7 for analysis) to “Strongly disagree” (1) with “Neither agree nor disagree” (4) at the midpoint.

Composite scores for each construct in Table 2 were then formed by averaging responses across all relevant items after reverse-scoring where necessary. Larger numbers refer to greater endorsement of a construct. Figure 3 shows the distributions of the average scores for the 8 constructs.

Analysis of the three items for the CRT revealed that participants on average achieved 0.47 correct (out of a possible 3). This mean is lower than what is typically observed, although mean and distribution of responses were commensurate with the lowest-performing sample reported by Frederick (2005). The percentage of participants who got 0, 1, 2, or 3 correct was 70.3, 16.8, 8.3, and 4.6, respectively. (Missing values arising from responses such as “unsure” or “I don’t know” were omitted from computation of each person’s total and were thus considered errors).

### Latent variable modeling

Our preregistered analysis plan identified structural equation modeling (SEM) as our principal analysis technique. We therefore sought to represent each construct by a latent variable that was estimated from the responses to the corresponding set of items. Latent variables are free of measurement error, and thus none of the estimated effects are attenuated by measurement error (Coffman & MacCallum, 2005). The analysis plan did not, however, specify particular SEM models, and our remaining analysis thus conformed to the analysis plan without being prescribed by it. All SEM was conducted using the *lavaan* package in R (Rosseel, 2012).

SEM models with more than 20 items overall are often too complex and unwieldy to achieve adequate levels of model fit (Bentler & Chou, 1987). One way to overcome this problem is by averaging the item scores measuring each construct into a single-indicator variable for SEM, a procedure known as item parceling. Averaging, however, may obscure multi-dimensionality (Little, Cunningham, Shahar, & Widaman, 2002). To retain the advantage of parceling without acquiring the problems arising from averaging, we first modeled each hypothesized latent variable using conventional SEM. These models used each construct’s respective items as a separate indicator variable and checked for the presence of multi-dimensionality.

#### Measurement models

To reduce potentially problematic differences in variance between the slider variables (range 0–100) and the remaining items, the slider results were rescaled to the range 1–7. All 8 constructs measured by the 7-point items exhibited an essentially uni-dimensional structure, except that in all cases a correlation between the residuals of two items had to be added to the single-factor model to achieve a satisfactory fit. Table 3 reports the fit statistics for those 8 measurement models. For the free-market and vaccination constructs that had been employed in previous SEM modeling (Lewandowsky, Gignac, & Oberauer, 2013), the fit statistics were similar and the correlated residuals involved the same items as before. All models fit well or extremely well, with the possible exception of the measurement model for evolution, one of whose fit indices was not satisfactory (RMSEA = 0.128).

**Table 3:** Model fit indices associated with the measurement models for all uni-dimensional constructs.

Construct	*χ*^2^	*df*	SRMR	CFI	RMSEA	90% CI	Correlated residuals

Free market	10.24	4	0.019	0.994	0.039	0.009–0.07	*FMUnresBest* ↔ *FMMoreImp*
Religiosity	41.93	4	0.026	0.984	0.097	0.071–0.124	*RelGod* ↔ *RelAfterlife*
Evolution	70.26	4	0.044	0.952	0.128	0.102–0.155	*EvoCreated* ↔ *EvoCrisis*
Vaccinations	14.21	4	0.017	0.995	0.05	0.024–0.079	*VaxNegSide* ↔ *VaxRisky*
Rejection of CAM	6.53	4	0.017	0.996	0.025	0–0.058	*CAMDanger* ↔ *CAMIneffect*
Men & women evolved differently	3.46	4	0.012	1	0	0–0.044	*MWEvoViol* ↔ *MWEvoNurture*
Men & women naturally different	9.31	4	0.018	0.993	0.036	0–0.067	*MWNatAggress* ↔ *MWNatCaring*
Men & women are the same	24.58	4	0.028	0.972	0.071	0.046–0.099	*MWEquDiff* ↔ *MWEquInvent*
Socio-political conservatism	281.49	33	0.045	0.939	0.086	0.077–0.095	*TradMarriage* ↔ *TradValues*
							*Military* ↔ *Patriotism*
CRT	14.39	2	0.072	0.982	0.078	0.044–0.118	N/A

Unlike for the other constructs, it proved impossible to create a unidimensional model for the 14 items of the socio-political conservatism scale. Inspection of the correlations among items revealed that items of different polarity correlated little with each other: that is, responses to the issues abortion, welfare, tax, and immigration correlated little with the remaining issues, even after reverse scoring.^3^ We resolved this difficulty in two ways: First, we focused exclusively on the 10 items with a conservative polarity to create a uni-dimensional measurement model. The penultimate row in Table 3 contains the fit statistic for this single-factor model, which had to be augmented with two pairwise correlations between residuals. Second, we created a composite score by averaging across all 14 items irrespective of polarity. We report an analysis based on composites for all constructs (cf. Figure 3) in parallel to the SEM modeling where appropriate (e.g., Table 6).

Finally, to represent CRT performance, we created a measurement model from the three binary indicators of performance, constraining the weights to be equal and imposing an ordinal level of measurement. The last row in Table 3 contains the fit statistics for this model.

#### Single-indicator latent variable models

We consider the performance of the unidimensional measurement models after addition of single pairwise correlations sufficient to establish the essentially unidimensional structure of our contructs. This conclusion is supported by the fact that the model fit ranged from good to excellent (Hooper, Coughlan, & Mullen, 2008) for most constructs. However, the conclusion comes with the qualification that for the evolution construct the fit was acceptable but not spectacular, and that two pairwise correlations were required for the socio-political conservatism construct (although this construct also comprised many more items, which justifies inclusion of a further pairwise correlation).

We proceeded with the construction of single-indicator latent variables (Hayduk, 1996; Jöreskog & Sörbom, 1982). In single-indicator models, each latent variable is defined by one indicator consisting of an equally-weighted composite of the items (i.e., the mean score). The true-score variance for each latent variable is then obtained by constraining the single-indicator’s error variance to: (1-reliability) × *s*^2^, where *s*^2^ is equal to the composite score’s total variance (Jöreskog & Sörbom, 1982).

An accurate estimator of reliability is *ω* (Komaroff, 1997; Raykov, 1997), which we estimated using the individual measurement models (Table 3; for details, see Raykov, 1997). The error variances of the indicators were set to the values shown in Table 4 and all remaining SEM models used the single-indicator latent variables thus defined. The present estimates of *ω* are in close agreement to the values observed by Lewandowsky, Gignac, and Oberauer (2013) for the constructs used by both studies (free market and vaccinations). When present in a model, CRT performance was represented by the latent variable defined by the three binary indicators as described above.

**Table 4:** Summary statistics of single-indicator latent variable models.

Construct	*s^a^*	*ω^b^*	(1-*ω*) × *s*^2^*^c^*

Free market	0.99	0.58	0.413
Religiosity	1.57	0.85	0.364
Evolution	1.17	0.69	0.423
Vaccinations	1.28	0.76	0.389
Rejection of CAM	0.76	0.4	0.344
Men & women evolved differently	0.94	0.56	0.394
Men & women naturally different	1.01	0.61	0.394
Men & women are the same	1.04	0.63	0.4
Socio-political conservatism	1.2	0.87	0.19

*^a^* Standard deviation of composite score. *^b^* √*ω* corresponds to the loading of a single-indicator manifest variable on its factor. *^c^* Error variance of each single-indicator latent variable.

#### Correlations among constructs

Table 5 shows the correlation matrix for the single-indicator latent variables (and the CRT latent variable). All correlations were significant at *p* < .01 or less, with the exception of those identified as “*ns*” in the table. The covariance matrix of latent variables for this solution was not positive definite, likely reflecting linear dependency between factors deriving from the high correlation between the two constructs probing differences between men and women (evolved differently vs. naturally different). To ensure that the ill-conditioned covariance matrix did not compromise the results, Table 6 shows the same correlations based on the composite scores instead of latent variables. As expected, those correlations are attenuated owing to measurement error compared to the correlations based on latent variables. However, their pattern is identical to that shown in Table 5, suggesting that the latent-variable model is adequately identified notwithstanding the ill-conditioned covariance matrix.

**Table 5:** Correlations among latent variables.

	Free market	Evolution	Rejection of CAM	Men/women evolved differently	Men/women naturally different	Men/women are the same	Religiosity	Vaccinations	Socio-political conservatism

Evolution	–0.389								
Rejection of CAM	–0.182	0.185							
Men/women evolved differently	0.179	0.327	0.048*ns*						
Men/women naturally different	0.426	–0.307	–0.020*ns*	0.822					
Men/women are the same	–0.271	0.331	–0.009*ns*	–0.350	–0.669				
Religiosity	0.288	–0.598	–0.199	–0.110	0.256	–0.246			
Vaccinations	–0.243	0.352	0.409	–0.043*ns*	–0.152	0.192	–0.062*ns*		
Socio-pol conservatism	0.484	–0.315	–0.199	0.069*ns*	0.400	–0.351	0.563	–0.008*ns*	
CRT	–0.206	0.269	0.183	–0.010*ns*	–0.035*ns*	–0.130	–0.201	0.197	–0.009*ns*

*Note*: Correlations identified with “ns” are non-significant, *p* > .10. All others are significant at *p* < .01 or less.

**Table 6:** Correlations among composite measures for all constructs.

	Free market	Evolution	Rejection of CAM	Men/women evolved differently	Men/women naturally different	Men/women are the same	Religiosity	Vaccinations	Socio-political conservatism

Evolution	–0.251								
Rejection of CAM	–0.082	0.086							
Men/women evolved differently	0.104	0.208	0.022*ns*						
Men/women naturally different	0.249	–0.185	–0.006*ns*	0.496					
Men/women are the same	–0.166	0.207	–0.006*ns*	–0.209	–0.407				
Religiosity	0.198	–0.454	–0.119	–0.087	0.167	–0.170			
Vaccinations	–0.159	0.255	0.220	–0.033*ns*	–0.108	0.122	–0.050*ns*		
Socio-pol conservatism	0.418	–0.319	–0.101	0.091	0.350	–0.338	0.520	–0.053*ns*	
CRT	–0.116	0.167	0.084	0.006ns	–0.008*ns*	–0.077	–0.144	0.136	–0.011*ns*

*Note*: Correlations identified with “ns” are non-significant, *p* > .10. All others are significant at *p* < .01 or less.

Because three of our constructs related to gender differences, we used participants’ gender as a grouping variable in two additional SEM models of the correlations among latent variables to examine whether men and women differed in their attitude structures. One model estimated all parameters independently for men and women. We compared this full model to two constrained models: in the first, partially constrained model we forced the covariances between the three gender-related constructs to be equal, and in the second, fully constrained model all covariances among latent variables were forced to be equal between groups. The partially constrained model fit as well as the full model, *χ*^2^(3) = 0.07, *p* < 0.9950. The fully constrained model, by contrast, incurred a significant loss of fit compared to the partially constrained model, *χ*^2^(42) = 96.88, *p* < 0.0001.

Because the associations among the gender related constructs did not differ with gender of the participants, and because the fully constrained model nonetheless fit well in absolute terms, SRMR = 0.043; CFI = 0.966; RMSEA = 0.042, CI: 0.031–0.052, we decided that for our purposes, the fully constrained model provided an adequate description of the data. We therefore do not consider the effects of participants’ gender further.

#### Predicting scientific attitudes

We next explored the relationship among the political worldview constructs, (socio-political conservatism, religiosity, and free-market endorsement), and how those constructs in turn would predict attitudes to the scientific themes (vaccinations, CAM rejection, and evolution).

Theoretical considerations and previous findings suggest that the three aspects of political worldview are differentially related to the three science themes: Rejection of evolution is mostly motivated religiously; concerning vaccinations, Lewandowsky, Gignac, and Oberauer (2013) found opposite-signed regression weights for free market (negative) and another measure of socio-political conservatism (positive). Therefore, we aimed to tease apart the contributions of general political orientation from its more specific aspects to science rejection in the three domains. Development of the following models was thus guided by prior theorizing and results, but also informed by the present data, and therefore they must be considered exploratory.

**Figure 4:**
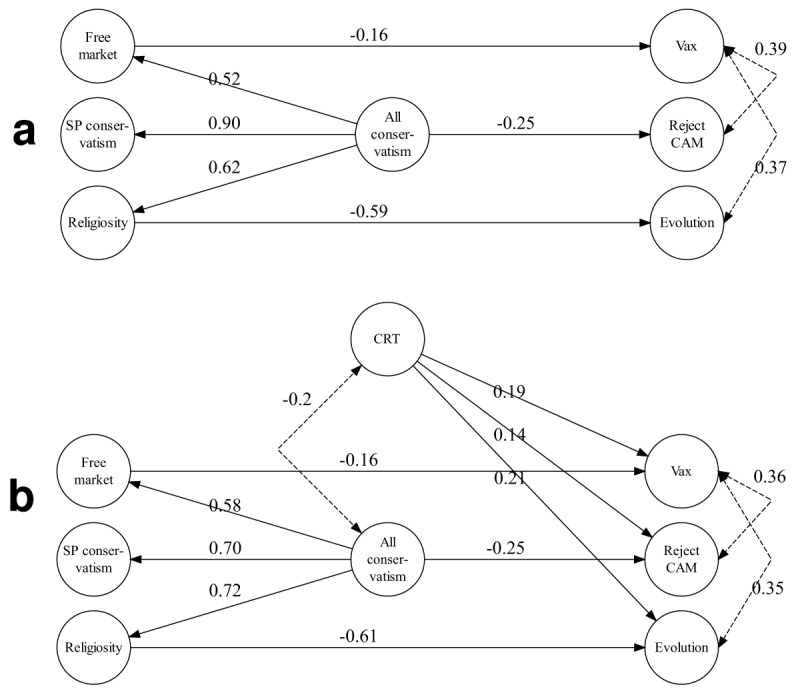
Structural equation model to predict scientific attitudes. Panel a shows the model with the three worldview constructs as predictors. Panel b shows the same model after inclusion of a factor representing CRT performance. All links and correlations shown are standardized and significant; all *p* ≤ .05. Dashed lines indicate correlations between latent variables. Indicator variables and their loadings, and disturbances on endogenous factors are not shown. Links between latent variables that are not shown are constrained to zero. Loadings and variances of single-indicator latent variables are reported in Tables 3 and 4.

We first created a hierarchical-factor model in which the three worldview constructs were used as indicators for an over-arching second-order factor (called “All conservatism”). The polarity of this factor was pointing towards increasingly conservative worldviews (i.e., greater endorsement of free markets, greater socio-political conservatism, and stronger religiosity). We used this second-order factor, in turn, to predict scientific attitudes, adding direct links from the first-order constructs where necessary, and constraining non-significant links to zero when possible without incurring loss of fit. The final political model obtained in this manner fit very well, SRMR = 0.037; CFI = 0.953; RMSEA = 0.073, CI: 0.053–0.094, and is shown in Figure 4 (panel a).

The model captures the correlations between the three scientific constructs (although unlike for the first-order correlations in Table 5, CAM rejection was not significantly correlated with evolution). This replicates other findings that people’s attitudes towards scientific issues often covary (e.g., Lewandowsky, Gignac, & Vaughan, 2013). Accordingly, one or more aspects of conservatism uniformly predicted reduced acceptance of scientific issues. The All conservatism factor was found to be negatively associated with rejection of CAM, suggesting that people with conservative worldviews are more likely to accept CAM. The All conservatism factor was not associated with the remaining two scientific themes. Instead, it was the sub-components of overall conservatism that predicted rejection of those other issues. Rejection of vaccinations is predicted exclusively by free-market endorsement and rejection of evolution by religiosity. This model shows that rejection of science is not simply driven by a general left-right dimension of political orientation. Our more differentiated assessment of worldview helped us identify relevant sub-dimensions of the overall cluster of worldviews, which differentially influence people’s acceptance or rejection of scientific propositions. Crucially, however, the polarity of the effects was uniform across issues, with no evidence that people on the political left were less inclined to accept scientific issues.

We next expanded this model by adding CRT performance as a further predictor but clamping the regression estimates to their values from the original model. This expanded model is shown in panel b of Figure 4 and, notwithstanding the imposition of strong constraints, it fit acceptably well, *χ*^2^(26) = 113.86; SRMR = 0.064; CFI = 0.951; RMSEA = 0.058 (90% CI: 0.047–0.069). The uniformly positive weights between CRT performance and endorsement of science again replicate earlier results (Shtulman & McCallum, 2014; Wagner-Egger, Delouvée, Gauvrit, & Dieguez, 2018) and confirm that the first-order correlations in Table 5 stand up to the inclusion of all worldview-related constructs.

#### Predicting attitudes towards gender equality

**Figure 5:**
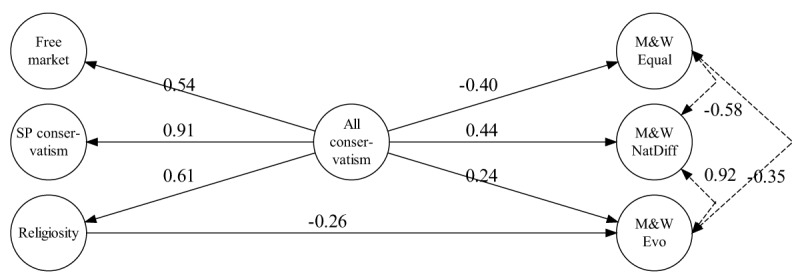
Structural equation model to predict gender attitudes from worldviews. All links and correlations shown are standardized and significant; all *p* ≤ .05. Dashed lines indicate correlations between latent variables. Indicator variables and their loadings, and disturbances on endogenous factors are not shown. Links between latent variables that are not shown are constrained to zero. Loadings and variances of single-indicator latent variables are reported in Tables 3 and 4.

We developed a parallel hierarchical model to predict attitudes towards the gender-related constructs from the same set of worldview constructs. The model is shown in Figure 5 and fit very well, *χ*^2^(5) = 20.45; SRMR = 0.032; CFI = 0.985; RMSEA = 0.055 (90% CI: 0.032–0.081). The model shows that the All conservatism factor predicted all aspects of gender attitudes in the expected direction: greater conservatism predicted less endorsement of the idea that men and women are equal, and it predicted greater acceptance of evolved or natural differences between men and women. In addition, religiosity uniquely predicted rejection of the idea that men and women evolved differently, thus again dissociating between different aspects of overall conservatism.

### Nurture vs. nature vs. evolution

**Figure 6:**
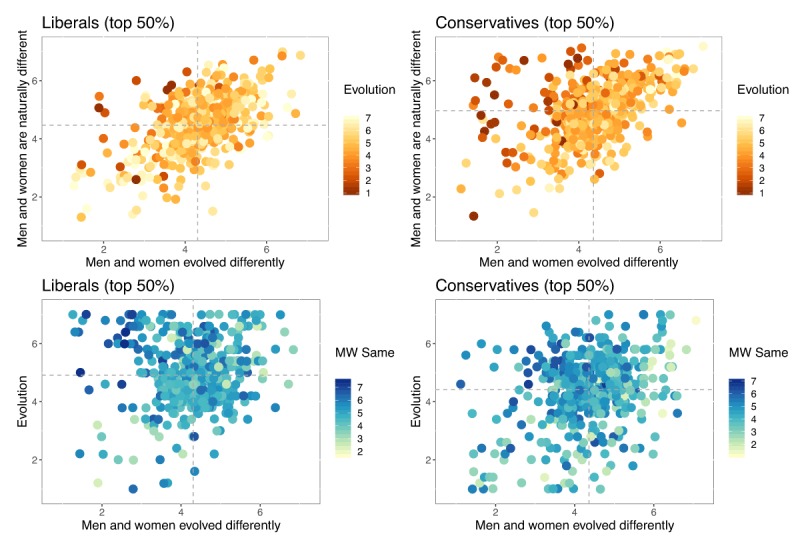
Relationship between the three gender-related constructs and acceptance of evolution. Liberals are shown on the left and conservatives on the right (median split of socio-political conservatism construct). The top panels show acceptance of evolution (represented by color of plotting symbols) and people’s responses to the constructs probing the origin of gender differences. The bottom panels show belief in gender equality (represented by color of plotting symbols) and people’s responses to the two constructs probing evolution generally (ordinate) and that men and women evolved differently (abscissa). Composite scores are used for all constructs. Dashed horizontal and vertical lines represent marginal means. Points are randomly jittered to reduce over-printing.

The final analysis considered the relationship between people’s acceptance of evolution and the constructs that probed the origins and extent of gender differences. This analysis therefore explored the dilemmas postulated in Figure 1. We divided participants according to their political views, conducting a median split of the composite scores on the socio-political conservatism scale. Figure 6 illustrates the results, using composite scores for all constructs, with liberals shown on the left and conservatives on the right.

The top row of panels illustrates the putative dilemma confronting conservatives. The panels plot the two constructs concerning the origin of gender differences (men and women evolved differently [abscissa] vs. men and women are naturally different [ordinate]). The color of plotting symbols shows attitudes towards evolution, with dark dots indicating rejection and light dots acceptance. Several observations can be made: First, the two constructs for the origins of gender differences are highly correlated irrespective of political views. Second, conservatives are more likely overall to think that men and women differ naturally than liberals. Third, as expected from the pattern of correlations in Table 6, more conservatives than liberals reject Darwinian evolution (more dark points on the right). Crucially, conservatives who reject evolution also tend to endorse gender differences—however, as revealed by the cluster of dark points in the top left, that endorsement mainly involves the idea that men and women are somehow “naturally” different, rather than the idea that they “evolved differently.” Conservatives thus resolved their dilemma by ascribing gender differences to unspecified natural factors while rejecting evolution both generally and specifically with respect to gender differences.

The bottom row of panels illustrates the putative dilemma facing liberals. The panels plot the two constructs probing evolution, with gender differences (men and women evolved differently) on the abscissa, and general evolution acceptance on the ordinate. The color of plotting symbols shows attitudes towards gender equality, with darker points representing greater endorsement of equality. It is clear that more liberals than conservatives endorse evolution and that liberals are more inclined to endorse gender equality (dots are generally darker in the left panel). Crucially, a large number of liberals accept evolution and gender equality—however, as revealed by the cluster of dark points in the top left, that endorsement is accompanied by a reduced belief in the evolution of gender differences. Liberals thus resolved their dilemma by rejecting the evolution of gender differences while embracing evolution generally together with equality.

## Discussion

### Relationship to previous results

Our results coordinate well with multiple precedents in the literature, which we take up for each of the constructs examined. Considering first religiosity, we replicated the substantial association between stronger religious beliefs and conservatism in the American population (Malka et al., 2012; Schlenker, Chambers, & Le, 2012). In our study this association generalized across a broadly-defined socio-political conservatism construct as well as a specific construct targeting endorsement of laissez-faire free-market economics. We also replicated the long-standing strong negative association between religiosity and acceptance of evolution (e.g., Ecklund, Scheitle, Peifer, & Bolger, 2017; Tom, 2018) and the modest negative association between religiosity and analytic thinking (i.e., CRT performance) reported previously (Jack, Friedman, Boyatzis, & Taylor, 2016; Shenhav, Rand, & Greene, 2012; Stagnaro, Ross, Pennycook, & Rand, 2019). Likewise, the correlations between religiosity and the gender constructs (e.g., Table 5) are consistent with previous reports that religiosity predicts sexism (Van Assche et al., 2019). Our results go beyond previous findings because our scales did not probe discriminatory sexism but the origin of presumed gender differences. We find that religiosity makes it less likely that people believe that gender differences have evolved.

The negative association between religiosity and CAM rejection is also unsurprising in light of previous research that has shown acceptance of CAM to be driven by intuitive thinking, paranormal beliefs, and ontological confusions (Lindeman, 2011). At least one of those variables (intuitive thinking) is also known to be a predictor of religiosity (e.g., Shenhav et al., 2012). The positive correlation between CAM rejection and acceptance of vaccinations replicates much previous research (e.g., Attwell, Ward, Meyer, Rokkas, & Leask, 2018; Browne, Thomson, Rockloff, & Pennycook, 2015; Bryden, Browne, Rockloff, & Unsworth, 2018; Ernst, 2002).

However, our findings concerning religiosity also deviate from aspects of other recent research (Rutjens, Sutton, & van der Lee, 2018). Unlike Rutjens et al., we found no evidence of a link between religiosity and rejection of vaccinations. Given that Rutjens et at. observed this link only in some of their studies and only for some measures of religiosity (mainly measures of religious orthodoxy), we are not concerned about this apparent departure from previous results. Indeed, in another recent as-yet unpublished study involving identical constructs, we did observe a negative association between vaccination and religiosity, suggesting that this relationship may well be real but is only observable in certain circumstances.

Turning to the associations involving CRT performance, the observed modest but significant negative correlation with religiosity replicates previous results (Gervais & Norenzayan, 2012; Shenhav et al., 2012; Stagnaro et al., 2019). Jost (2017) reported a meta analysis of 13 studies that related CRT performance to political views. The vast majority of those studies showed that liberals exhibited more cognitive reflection than conservatives. In the present data, this is echoed by the modest negative correlation with free market, although it was not reflected in the socio-political conservatism measure. The positive associations of the CRT with endorsement of all three scientific constructs, vaccination, CAM rejection, and evolution replicate similar previous findings (Shtulman & McCallum, 2014; Wagner-Egger et al., 2018). The association also coordinates well with recent findings that analytical thinking is associated with better differentiation between “fake news” and valid information (Pennycook & Rand, 2018).

### Rejection of science on the political left?

Our findings provide little or no evidence that people on the political left reject vaccinations. On the contrary, to the extent that worldviews determined vaccination attitudes, it was free-market endorsement that predicted rejection. This result parallels a similar association observed by Hornsey, Harris, and Fielding (2018), albeit using a different instrument to measure Libertarian attidudes (hierarchical-individualism as opposed to free-market endorsement). The result is also consonant with the notion that libertarians object to the government intrusion arising from mandatory vaccination programs (Kahan et al., 2010). It also meshes well with the pattern observed by Lewandowsky, Gignac, and Oberauer (2013), who showed that when socio-political conservatism was removed from a model, free-market endorsement on its own predicted rejection of vaccinations (whereas the converse was not true). Overall, our results thus converge with other recent findings that have found an association between right-wing politics and rejection of vaccinations (Baumgaertner, Carlisle, & Justwan, 2018; Kahan et al., 2010; Rabinowitz et al., 2016). In a recent cross-sectional analysis of voting behavior and vaccination rates across European countries, Kennedy (2019) found a strong relationship between the vote share for populist parties and vaccine hesitancy.

Similarly, contrary to reports that CAM use and left-wing ideas have a natural affinity for each other (see, e.g., Keshet, 2009), we found that CAM rejection was negatively, but modestly, associated with the All conservatism factor that subsumed all three of our worldview constructs; namely, religiosity, free market endorsement, and socio-political conservatism. Moreover, in our data, none of the gender constructs were associated with CAM attitudes. This runs counter to the idea that CAM use is “feminist” (Scott, 1998). To our knowledge, our results constitute the first empirical examination of the links between political views and CAM attitudes. Our results that conservatives are more likely to embrace CAM is consonant with historical analyses that have found strong links between right-wing organizations, such as the John Birch Society in the U.S., and endorsement of “alternative” cancer treatments (Markle, Petersen, & Wagenfeld, 1978). The present result adds to the list of failed attempts to discover science denial on the political left (e.g., Hamilton, 2011, 2015; Hamilton, Hartter, & Saito, 2015; Hamilton, Hartter, Lemcke-Stampone, et al., 2015; Kahan et al., 2010; Lewandowsky, Gignac, & Oberauer, 2013; Tom, 2018).

### Attitudes towards gender differences

We observed an intriguing interplay of the attitudes towards general Darwinian evolution, gender differences, and how those gender differences might have arisen. At a coarse level of analysis, we observed three unsurprising associations: The idea that men and women differ naturally was highly correlated with the idea that they evolved differently, but was negatively correlated with the construct that proclaimed gender equality. The equality construct was also negatively correlated with the idea that men and women evolved differently, although that correlation was smaller than for natural differences.

At a more detailed level of analysis, several intriguing associations emerged. First, acceptance of general Darwinian evolution was positively associated with two seemingly conflicting constructs; namely, that men and women evolved differently *and* that they are the same. Moreover, evolution was negatively correlated with the idea that men and women are naturally different, even though evolution is one way in which such “natural” differences might have emerged. A similarly nuanced pattern obtained when the worldview constructs were used to predict gender attitudes. Although the over-arching All conservatism factor functioned as expected, with negative weights for gender equality and positive weights for the two constructs insisting on gender differences, there was an additional selective effect of religiosity on the rejection of evolved gender differences.

Further analysis revealed that the involvement of evolution, either on its own or in explaining gender differences, served as a “wedge issue” that disrupted otherwise straightforward associations between right-wing politics and opposition to gender equality (and, vice versa, rejection of gender differences and left-wing politics) and—as foreshadowed in Figure 1—created dilemmas for participants of all political persuasions. As noted in connection with Figure 6, conservatives who strongly rejected Darwinian evolution resolved their dilemma by endorsing “natural” gender differences while rejecting evolved gender differences. Those participants were thus willing to forego endorsement of gender differences to maintain consistency with their opposition to evolution. Conversely, liberals who are strongly committed to gender equality tended to reject the idea of evolved gender differences, even though those participants were demonstrably committed to accepting evolution. Those participants were thus willing to forego endorsement of a specific manifestation of evolution to maintain consistency with their commitment to equality. Thus, partisans of either stripe can agree in their rejection of the idea that men and women evolved differently, but they do so for entirely different reasons. Conservatives do so when they are committed to reject evolution, and liberals do so when they are committed to gender equality. Both groups therefore resolve the dilemmas posed by our gender constructs by “sacrificing” endorsement of evolved gender differences.

## Conclusion

Our results contribute to two seemingly conflicting streams of outcomes in the literature on how worldviews moderate people’s responses to scientific issues. On the one hand, there is much evidence for pervasive attitudinal asymmetry, at least in the United States, with conservatives being more likely to reject well-established scientific propositions than liberals. To date, little or no evidence for left-wing science denial has been reported. We add to this stream by showing that, contrary to previous largely anecdotal reports, liberals are more likely to reject complementary and alternative medicines, in line with the scientific evidence, than conservatives.

On the other hand, there is considerable evidence that liberals and conservatives *process* scientific data in a symmetrical fashion. That is, liberals and conservatives alike resort to the same cognitive shortcuts when data conform to their biases, giving rise to a symmetric set of errors (Kahan, Peters, Dawson, & Slovic, 2017; Washburn & Skitka, 2018). We also add to this stream of research by showing that, when confronted by worldview-triggered dilemmas, both liberals and conservatives resolve those dilemmas in an equally “rational” fashion, by selectively “sacrificing” endorsement of a specific construct about gender differences. Liberals, who generally endorse evolution, believe that for some reason it did not affect differences between the sexes; this could be rationalized perhaps by assuming that evolution causes differences only between but not within species. Conservatives, who frequently reject evolution, believe that men and women differ naturally without having evolved differently; this could be rationalized by assuming, for instance, that those natural differences were the result of divine intervention.

A final contribution of our study is that it points to the advantages of a more nuanced analysis of political worldviews, beyond a convenient but simplistic classification of people into left and right, or liberals and conservatives. While this classification is sufficient to explain some scientific attitudes—for example, it matters little how one measures political worldviews to explain rejection of climate science (e.g., Hornsey, Harris, Bain, & Fielding, 2016; Kahan, 2015)—there are other circumstances in which a more nuanced differentiation between different aspects of worldviews provides considerably greater explanatory power.

## Data Accessibility Statements

All data and code is publicly available at the links provided in the article.

## Footnotes

Here we are concerned only with attitudes towards scientific issues, as revealed in surveys. We are not concerned with laboratory experiments involving synthetic stimuli, which have often shown ideological symmetry in people’s reliance on cognitive shortcuts (e.g., Washburn & Skitka, 2018).

Nothwithstanding the ostensibly evolutionary constraints on mate choice illustrated by Buss (1989), subsequent research has shown that the preferences (e.g., for men who “provide” for women) are actually related to nations’ gender parity (Zentner & Mitura, 2012).

Everett (2013) reported a two-factor solution for his scale and the difficulties of fitting a unidimensional model are thus not unexpected. However, the correlational pattern observed here differed from that reported by Everett.
